# Autophagy-related protein LC3β and its association with clinical-pathological characteristics, mismatch repair proteins and survival in colorectal carcinoma

**DOI:** 10.3389/fmed.2025.1512127

**Published:** 2025-02-13

**Authors:** Samuel Gakinya, Ancent K. Nzioka, Alex G. Mugo, Timothy Onyuma, James Ogutu

**Affiliations:** ^1^Department of Pathology, Aga Khan University, Nairobi, Kenya; ^2^Department of Pathology, Kenyatta University Teaching, Referral and Research Hospital, Nairobi, Kenya; ^3^Department of Pathology, Kenyatta National Hospital, Nairobi, Kenya; ^4^Department of Microbiology, Kenyatta University, Nairobi, Kenya

**Keywords:** LC3β, autophagy, colorectal carcinoma, mismatch repair (MMR), Kenya, clinical-pathologic characteristics

## Abstract

**Introduction:**

Autophagy is a metabolic process that serves to maintain cellular homeostasis as well as enable the cell to adapt to metabolic stress. In malignant cells, autophagy has been associated with drug resistance, metastasis and poor outcome. Colorectal carcinoma is a leading cause of cancer morbidity and mortality worldwide. The management and outcome are dependent on the tumor clinical and pathological characteristics. Autophagy is a potential therapeutic target as well as prognostic biomarker given its role in cancer pathogenesis. This study aimed at evaluating the autophagy status of colorectal carcinomas for tumors diagnosed at the Aga Khan University Hospital, Nairobi and establish its association with clinical-pathological characteristics including age, tumor location, tumor grade, tumor pathological stage, tumor nodal stage, tumor budding, tumor-infiltrating lymphocytes (TILs), Mismatch repair protein status (MMR), HER2 status and patient survival.

**Methods:**

The study assessed the autophagy status of 114 colorectal carcinoma cases using immunohistochemistry for autophagy related protein LC3β. The clinical-pathological characteristics were determined by examining the medical records and evaluation of hematoxylin and eosin-stained slides. HER2 and MMR status were evaluated using immunohistochemistry. The treatment outcome was determined from the patient's records by checking for date of last visit or death.

**Results and discussion:**

The mean age of patients in our study was 58years. There were more males 61.8% (*n* = 70) than females 38.6% (*n* = 44). Most of the patients had high pathological tumor stage of pT3 and pT4. Majority of the tumors showed intermediate tumor budding and weak tumor-infiltrating lymphocytes. The mismatch repair deficiency and HER2 overexpression were found in 14.9% (*n* = 17) and 2.6% (*n* = 3) of the cases respectively. LC3β was overexpressed in 36% (*n* = 41) of the cases and was significantly more common in females (*p* = 0.013). The LC3β status showed no significant association with age, tumor location, tumor grade, tumor stage, nodal stage, tumor budding, tumor-infiltrating lymphocytes, MMR status, HER2 status or patient survival. Future prospective studies are recommended to further explore the utility of autophagy as a prognostic and predictive biomarker.

## Introduction

Autophagy is an essential catabolic mechanism that helps to sustain eukaryotic homeostasis as well as help them adapt to varying metabolic stress (1). Three different types of autophagy are described: macroautophagy, microautophagy, and chaperone-mediated autophagy. This study focuses on macroautophagy (hereon referred to as autophagy) which is characterized by the engulfment of organelles in an autophagolysosome where degradation occurs.

Autophagy develops through a series of steps which include induction, nucleation, elongation, maturation, and degradation (2). Induction occurs following inhibition of mammalian Target of Rapamycin (mTOR) and results in nucleation, the formation on the endoplasmic reticulum of a double lipid membrane phagophore. This then elongates and recruits the “cargo” which is composed of the organelles to be degraded. The phagophore then closes to form the autophagosome. The autopohagosome fuse with lysosome to form autophagolysosome where the cargo is degraded and products released into the cytoplasm for recycling.

This process is controlled by numerous proteins collectively called autophagy-related proteins (ATG). One such protein is microtubule light chain 3β [LC3β; (3, 4)]. LC3β is localized in the nucleus and cytoplasm. Upon stimulation, there is promotion of transfer LC3β from the nucleus to the cytoplasm where it forms part of the autophagy core machinery (5). LC3β is then activated and the activated LC3β is attached on the inner and outer layer of the autophagosome right from the early stage of phagophore to the late stage of autophagolysosome formation. As a result, expression and localization of this protein has been used as a surrogate for assessing autophagy activity in the cell.

While in normal cells autophagy serves an important physiologic and survival function, in malignant cells autophagy has been associated with promotion of cell survival, metastasis, drug resistance and tumor recurrence all of which are associated with poor prognosis (6, 7). These are achieved through several mechanisms which include increased tolerance to metabolic stress, induction of dormancy, inhibition of apoptosis and promotion of metastasis via induction of epithelial mesenchymal transition. Autophagy is therefore a potential candidate as a target for cancer therapy as well as a prognostic marker.

Colorectal carcinoma is a leading cause of cancer morbidity and mortality worldwide. It is the third most common cancer diagnosis with an incidence of 10%. The incidence is higher in the developed countries (8). In Kenya, the incidence is 3.7% and 2.3% for colon and rectal cancer, respectively (9). The mainstay of management of colorectal carcinoma is surgical excision with or without chemotherapy, radiotherapy and targeted therapy. The type and outcome of treatment is dependent on pathological characteristics which include, tumor stage and grade, tumor-infiltrating lymphocytes, tumor budding and biomarker status (10–13). Among the established biomarkers in colorectal carcinoma include microsatellite stability/mismatch repair (MSI/MMR) status and HER2 amplification (11, 14). These biomarkers are of prognostic and predictive significance.

Given the role of autophagy in the pathogenesis of cancer, several studies have been conducted to investigate its potential as a prognostic and predictive biomarker. In their study, Sato et al. (15) showed that amino acid deprivation in colon cancer cell lines led to induction of autophagy and development of tolerance to nutrient deprivation. Inhibition of autophagy in the same cell lines resulted in enhanced apoptosis. These results suggest that autophagy is an essential survival mechanism for colorectal carcinoma in the harsh nutrient deprived tumor microenvironment (15). Other studies have looked at the prognostic significance of autophagy in colorectal carcinoma with varying conclusions. In their study, Koustas et al. (16) showed upregulation of autophagy as assessed by expression autophagy protein Beclin 1 on immunohistochemistry was associated with poor overall survival and progression free survival (16). Other studies have used autophagy genes expression profile as a marker of autophagy and have identified profiles which are of prognostic significance (17, 18).

This study aimed at assessing the autophagy status in colorectal carcinoma and its association with clinical-pathological characteristics and survival in patients at the Aga Khan University hospital Nairobi. We determined the autophagy status, the clinical-pathological characteristics i.e., age, gender, tumor location, tumor histological type and grade, tumor pathological stage, tumor nodal stage, tumor-infiltrating lymphocytes, tumor budding, mismatch repair protein (MMR) deficiency and HER2 status and survival.

## Methods

The study was carried out at the Aga Khan University Hospital, a tertiary hospital in Kenya. It included 114 colorectal carcinoma resection specimens for patients treated during the period 2011 to 2022 identified from the laboratory information system.

The information on age, gender and tumor location was extracted from the patient's records. Assessment of tumor histological type and grade, tumor pathological stage, tumor nodal stage, tumor budding, tumor- infiltrating lymphocytes (TILs) and immunohistochemistry was done through examination of hematoxylin and eosin-stained slides by the first and second authors who are anatomic pathologists. The tumor grading, staging and budding were performed as described in the CAP Protocol for the Examination of Resection Specimens from Patients with Primary Carcinoma of the Colon and Rectum Version: 4.2.0.0 (19).

The tumor-infiltrating lymphocytes (TILs) were categorized into four as described by Klintrup et al. (20); 0 None (absence of reaction), 1 weak (mild and patchy increase of inflammatory cells with no destruction of malignant cells), 2 moderate (inflammatory cells formed a band-like infiltrate at the invasive margin with some destruction of cancer cell islets by inflammatory cells, and 3 severe (prominent inflammatory reaction, forming a cup-like zone at the invasive margin, and destruction of cancer cell islets).

The evaluation of autophagy (LC3β), MMR and HER2 was done using immunohistochemistry on tissue microarray. The tissue microarray recipient blocks were constructed from formalin-fixed, paraffin-embedded tissue blocks of the study samples. Briefly, the representative area was marked on hematoxylin and eosin-stained slides and the corresponding area punched from the tissue block and transferred to the recipient block. Each sample was duplicate. The examination and reporting of the stained slides was done independently by the first and second author after a joint session in which the interpretation parameters were agreed. In case of discrepancy, the slides were reviewed jointly for a consensus.

Immunohistochemistry was carried out using specific antibodies to LC3β (Medaysis, 1:00 dilution), MLH1 (DAKO, RTU), PMS2 (DAKO, RTU), MSH2 (DAKO, RTU), MSH6 (DAKO, RTU), HER2 (DAKO, 1:1200 dilution). Immunohistochemical staining for LC3β, HER2, MLH1, PMS2, MSH6 and MSH2 performed on 4-microns sections cut from the tissue microarray blocks using Dako auto stainer link-48 according to manufacturer's protocol. Briefly, tissue sections were mounted on super frost glass slides, deparaffinized and rehydrated through xylene and serial alcohol solutions. Retrieval was done by immersing the slides in citrate buffer PH 9.0 at 95°C for 20 min. The sections were treated with 0.3%hydrogen peroxide for 5 min to block endogenous peroxidase activity then incubated with primary antibody for 20 min. For LC3β, MSH2, and MLH1 they were further incubated with secondary antibody (mouse Linker) for 15 min. The sections were labeled using polymer flex/HRP added for 20 min, and the color reaction completed by FLEX DAB + chromogen hematoxylin. The sections were dehydrated in ascending series concentration of alcohols, cleared in xylene and mounted with DPX.

Interpretation of the LC3β status was as described in a previous study (21). Briefly, the status was determined by assessing the percentage of positively stained cells and the staining intensity. The percentage of positively stained cells was graded as: 0, ≤ 5%; 1, 6–35%; 2, 36–65%; and 3, 66–100%. The staining intensity was graded as: 0, no staining; 1, buff; 2, yellow; and 3, brown. The final staining score was calculated by multiplying the above-obtained scores. Tumors with an immunoreactive score of 0–3 were designated as negative, whereas those with 4–9 were classified as positive. There was agreement in 90.4% (*n* = 103) of the cases between the first and second authors upon independent reporting. Consensus was sought for the discrepant cases.

The assessment for MMR and HER2 was as described by The College of American Pathologists (CAP) Template for Reporting Results of Biomarker Testing of Specimens from Patients with Carcinoma of the Colon and Rectum Version: 1.3.0.0 (22). Briefly, tumors were categorized as having mismatch repair deficiency (MMRD) if loss of at least one MMR proteins (MLH1, PMS2, MSH2, MSH6) was detected and as HER2 positive if more than 50% of tumor cells showed an intense circumferential, basolateral, or lateral membrane staining.

The patient's survival was determined from the medical records by determining the date of last appointment post-surgery or date of death. Patients with no follow-up records were not included in the analysis on survival.

Statistical analyses were performed using IBM SPSS Statistics, Version 25 (Chicago, IL, USA). Comparative baseline characteristics for the participants were summarized in tables with mean and standard deviations (SD) calculated for continuous numerical variables, while frequency and percentages were calculated for categorical variables. The Chi-square test, and Fisher's exact test were used for the analysis to establish an association between the immunohistochemical expression and the clinicopathological variables. Overall survival (OS) in months was calculated from the date of diagnosis to the date of death or the last follow-up. Survival analysis for available data was evaluated by the Kaplan-Meier method, and significant differences between the LC3β and MMR groups were identified by the log-rank test.

Ethical clearance was given by the Aga Khan University Nairobi, Institutional Scientific and Ethics Review Committee (ISERC) and NACOSTI research permit was obtained. All patients' identities were anonymized, and the files were password protected.

## Results

### Baseline clinical-pathological characteristics and their association with LC3β expression

The baseline characteristics and their association with LC3β status are as shown in Table 1. Briefly, the study included 61.4% (*n* = 70) males and 38.6% (*n* = 44) females, with a mean age of 58 years. 94.7% (*n* = 108) of the tumors were adenocarcinoma NOS and 92.1% (*n* = 105) were well/moderately differentiated. 66.7% (*n* = 76) of the individuals were diagnosed with T3 stage tumors and 55.3% (*n* = 63) had N1 and N2 nodal involvement. The right colon was the most common location with 53.5% (*n* = 61) of the cases. Tumor budding was found to be mostly intermediate with 43% (*n* = 49) of the cases while tumor-infiltrating lymphocytes (TILs) was mainly weak with 53.5% (*n* = 61) of the cases. Mismatch repair deficiency (MMRD) was present in 14.9% (*n* = 17) of the cases with 76.4% (*n* = 13) of the cases being male. Deficiency in PMS2/MLH1 genes was the most common cause of mismatch repair deficiency with a frequency of 70.8% (*n* = 12). HER2 positivity was identified in 2.6% (*n* = 3) cases. LC3β expression was observed in 36% (*n* = 41) of the cases.

**Table 1:** Baseline clinical-pathological characteristics and association of LC3β status with gender, age, tumor stage, tumor location, nodal stage, histology type, tumor grade, tumor budding, TILs, MMR status, and HER2 status.

	***n* = 114**	**LC3β**	**LC3β**	**p-value**
	**N/Mean (%/SD)**	**Positive, N (% within group)**	**Negative, N (% within group)**	
**Gender**				
Male	70 (61.4)	19 (27.1)	51 (72.9)	**0.013**
Female	44 (38.6)	22 (50.0)	22 (50.0)	
**Age**				
Mean (SD)	57.64 (±14.28)			
≤ 60	62 (54.4)	22 (35.5)	40 (64.5)	0.907
>60	52 (45.6)	19 (36.5)	33 (63.5)	
**Tumor stage**				
T1	2 (1.8)	2 (100)	0 (0)	0.141
T2	9 (7.9)	5 (55.6)	4 (44.4)	
T3	76 (66.7)	25 (32.9)	51 (67.1)	
T4	27 (23.7)	9 (33.3)	18 (66.7)	
**Tumor location**				
Right colon	61 (53.5)	23 (37.7)	38 (62.3)	0.917
Left colon	50 (43.9)	17 (34.0)	33 (66.0)	
Rectum	3 (2.6)	1 (33.3)	2 (66.7)	
**Nodal stage**				
N0	51 (44.7)	20 (39.2)	31 (60.8)	0.628
N1	37 (32.5)	11 (29.7)	26 (70.3)	
N2	26 (22.8)	10 (38.5)	16 (61.5)	
**Tumor histological type**				
Adenocarcinoma NOS	108 (94.7)	39 (36.1)	69 (63.9)	0.311
SCC	1 (0.9)	1 (100)	0 (0.0)	
Mucinous adenocarcinoma	5 (4.4)	1 (20.0)	4 (80.0)	
**Tumor grade**				
Well/moderate	105 (92.1)	37 (35.2)	68 (64.8)	0.581
Poor/undifferentiated	9 (7.9)	4 (44.4)	4 (55.6)	
**Tumor budding**				
Low	42 (36.8)	15 (35.7)	27 (64.3)	0.365
Intermediate	49 (43)	15 (30.6)	34 (69.4)	
High	23 (20.2)	11 (47.8)	12 (52.2)	
**Tumor infiltrating lymphocytes (TILs)**				
None	40 (35.1)	17 (42.5)	23 (57.5)	0.117
Weak	61 (53.5)	17 (27.9)	44 (72.1)	
Moderate	13 (11.4)	7 (53.8)	6 (46.2)	
Severe	0 (0)	0	0	
**MMR status**				
MMRD	17 (14.9)	4 (23.5)	13 (76.5)	0.247
MMRS	97 (85.1)	37 (38.1)	60 (61.9)	
**HER2 status**				
Positive	3 (2.6)	1 (33.3)	2 (66.7)	0.923
Negative	111 (97.4)	40 (36.0)	71 (64.0)	
**LC3**β **status**				
Positive	41 (36)			
Negative	73 (64)			

Bold values represent p values less than 0.05.

LC3β positivity was significantly higher in females with a frequency of 50% (*n* = 22) compared to males' 27.1% (*n* = 19), with a *p*-value of 0.013. Age, tumor stage, tumor location, nodal stage, MMR status, and HER2 status showed no significant differences between LC3β positive and LC3β negative groups. Tumor stage distribution showed no significant differences, with T3 being the most common stage in both groups. Tumor location was similarly distributed between right colon, left colon, and rectum for both LC3β positive and LC3β negative groups. Nodal stage, MMR status, and HER2 status were also not significantly different between the groups, suggesting these factors were not associated with LC3β status.

**Figure 1:**
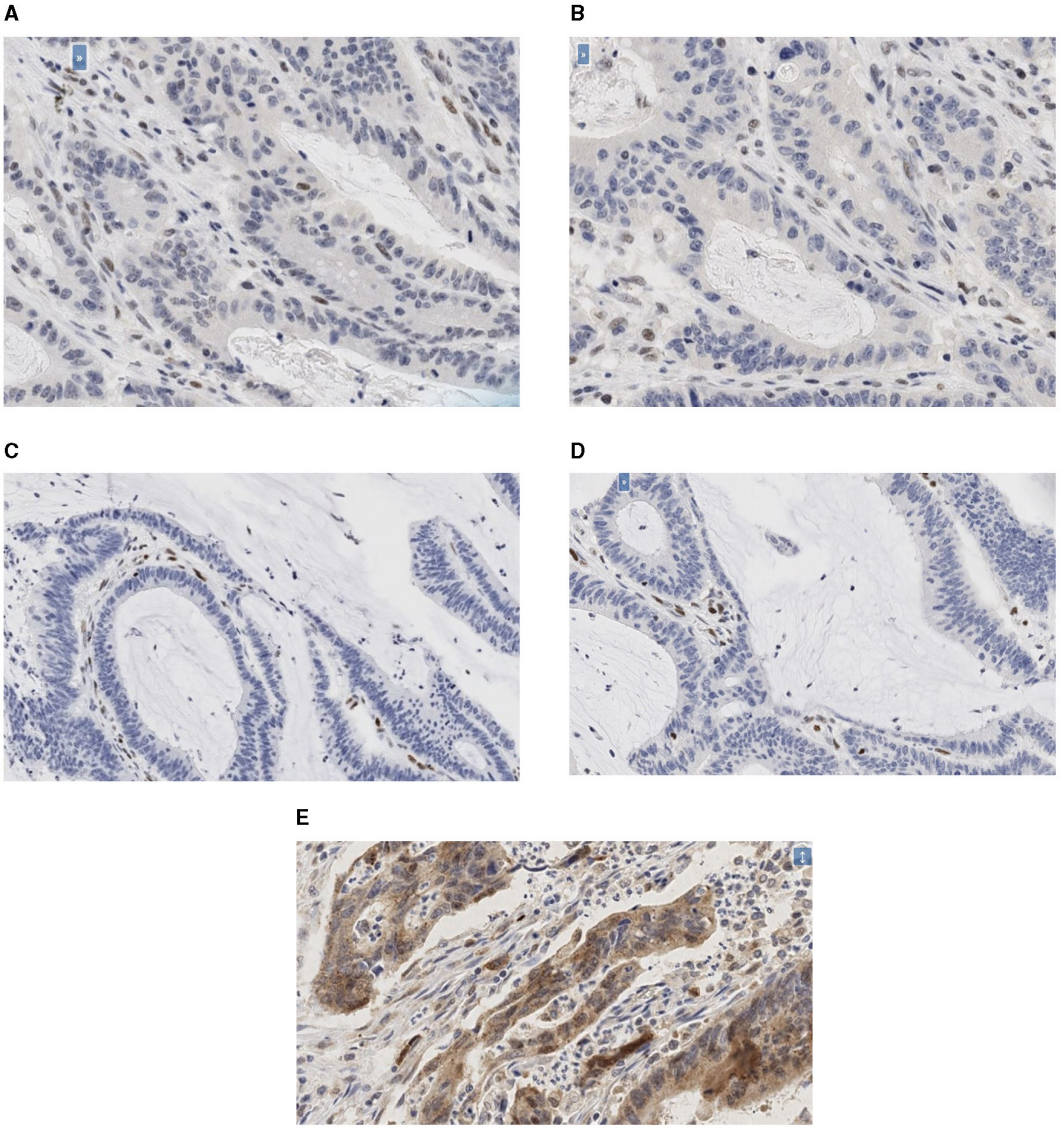
**(A–E)** Photomicrographs of the immunohistochemistry slides. **(A)** Photomicrographs of the immunohistochemistry slides of PMS2 deficient tumor (20X). **(B)** Photomicrographs of the immunohistochemistry slides of MLH1 deficient tumor (20X). **(C)** Photomicrographs of the immunohistochemistry slides of MSH2 deficient tumor (20X). **(D)** Photomicrographs of the immunohistochemistry slides of MSH6 deficient tumor (20X). **(E)** Photomicrographs of the immunohistochemistry slides of LC3β positive tumor (20X).

The photomicrographs of the immunohistochemistry slides for the PMS2, MLH1, MSH2, and MSH6 deficient tumors and LC3β positive tumor are as shown (Figures 1A–E).

### MMR status by gender, age, tumor stage, tumor location, nodal stage, histology type, tumor grade, tumor budding, TILs, LC3β status, and HER2 status

Table 2 compares the distribution and associated characteristics between individuals with mismatch repair deficient (MMRD) and mismatch repair stable (MMRS) status. It found no significant differences in gender distribution, age distribution, tumor stage distribution, or tumor location. Tumor budding (TB) did not show a significant difference between MMRD and MMRS groups (*p* = 0.406). Tumor-infiltrating lymphocytes (TILs) exhibited a significant association with MMRD status (*p* = 0.003), with a higher frequency of infiltrating lymphocytes in deficient than stable tumors. While MMRD individuals had a slightly higher prevalence of right colon with a frequency of 18% (*n* = 11), this wasn't statistically significant. There was a trend toward more N0 stage in MMRD cases, but not statistically significant. HER2 positive tumors were all MMRS, there was however so statistically significant difference between the two groups (Table 2).

**Table 2:** Frequency of MMR status by gender, age, tumor stage, tumor location, nodal stage, histology type, tumor grade, tumor budding, TILs, LC3β status, and HER2 status.

	***n* = 114**	**MMRD**	**MMRS**	**p-value**
	**N (%)**	**N (% within group)**	**N (% within group)**	
**Gender**				
Male	70 (61.4)	13 (18.6)	57 (81.4)	0.167
Female	44 (38.6)	4 (9.1)	40 (90.9)	
**Age**				
≤ 60	62 (54.4)	11 (17.7)	51 (82.3)	0.354
>60	52 (45.6)	6 (23.1)	20 (76.9)	
**Tumor stage**				
T1	2 (1.8)	0 (0)	2 (100)	0.919
T2	9 (7.9)	1 (11.1)	8 (88.9)	
T3	76 (66.7)	12 (15.8)	64 (84.2)	
T4	27 (23.7)	4 (14.8)	23 (85.2)	
**Tumor location**				
Right colon	61 (53.5)	11 (18.0)	50 (82.0)	0.515
Left colon	50 (43.9)	6 (12.0)	44 (88.0)	
Rectum	3 (2.6)	0 (0)	3 (100)	
**Nodal stage**				
N0	51 (44.7)	11 (21.6)	40 (78.4)	0.114
N1	37 (32.5)	5 (13.5)	32 (86.5)	
N2	26 (22.8)	1 (3.8)	25 (96.2)	
**Tumor histological type**				
Adenocarcinoma NOS	108 (94.7)	16 (14.8)	92 (85.2)	0.870
SCC	1 (0.9)	0 (0)	1 (100)	
Mucinous adenocarcinoma	5 (4.4)	1 (20.0)	4 (80.0)	
**Tumor grade**				
Well/moderate	105 (92.1)	16 (15.2)	89 (84.8)	0.739
Poor/undifferentiated	9 (7.9)	1 (11.1)	8 (88.9)	
**Tumor budding**				
Low	42 (36.8)	7 (41.2)	35 (36.1)	0.406
Intermediate	49 (43)	5 (29.4)	44 (45.4)	
High	23 (20.2)	5 (29.4)	18 (18.6)	
**Tumor infiltrating lymphocytes (TILs)**				
None	40 (35.1)	3 (7.5)	37 (92.5)	**0.003**
Weak	61 (53.5)	8 (13.1)	53 (86.9)	
Moderate	13 (11.4)	6 (46.2)	7 (53.8)	
Severe	0 (0)	0	0	
**LC3**β **status**				
Positive	41 (36.0)	4 (9.8)	37 (90.2)	0.247
Negative	73 (64.0)	13 (17.8)	60 (82.2)	
**HER2 status**				
Positive	3 (2.6)	0 (0)	3 (100)	0.462
Negative	111 (97.4)	17 (15.3)	94 (84.7)	

Bold values represent p values less than 0.05.

### Survival analysis with respect to LC3β status, MMR status, tumor and nodal stage

Of the 114 patients, survival data was obtainable for 59.

**Figure 2:**
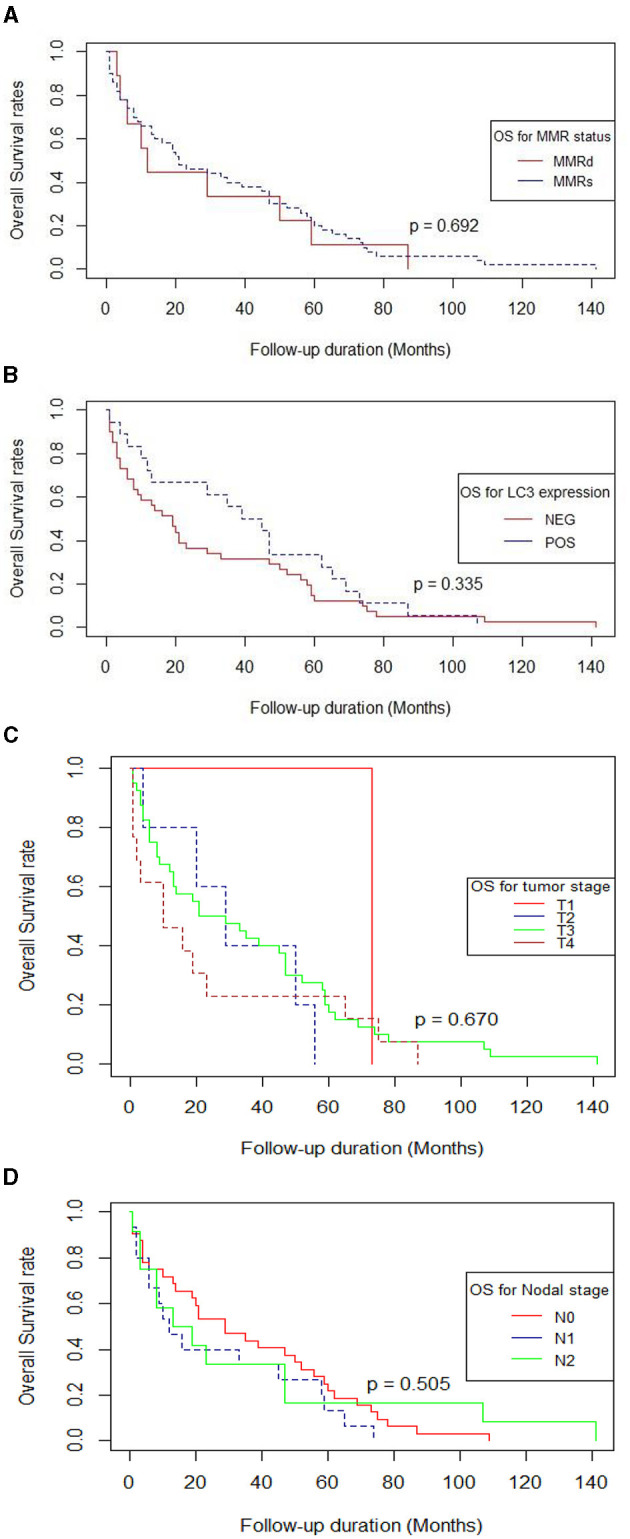
**(A–D)** Survival curves using the Kaplan-Meier method by log-rank test (*n* = 59). **(A)** MMR status, **(B)** LC3β status, **(C)** Tumor stage, **(D)** Nodal stage. **(A)** Survival curve using the Kaplan-Meier method by log-rank test for MMR. **(B)** Survival curve using the Kaplan-Meier method by log-rank test for LC3β. **(C)** Survival curve using the Kaplan-Meier method by log-rank test for Tumor stage. **(D)** Survival curve using the Kaplan-Meier method by log-rank test for Nodal stage.

Kaplan-Meier survival analysis was conducted to assess overall survival (OS) in months based on MMR status, LC3β status, tumor stage and nodal stage, with differences evaluated using the log-rank test (Table 3, Figures 2A–D).

**Table 3:** Kaplan-Meier survival analysis identified by the log-rank test with respect LC3β status, MMR status, tumor and nodal stage.

**Biomarker**	***n* = 59**	**OS in months Median [95% confidence intervals (CIs)]**	**p-value**
**LC3**β			
Positive	18	39 (18.21–59.79)	0.335
Negative	41	19 (10.24–27.76)	
**MMR status**			
MMRD	9	12 (6.15–17.84)	0.692
MMRS	50	21 (8.14–33.85)	
**Tumor stage**			
T1	1	73 (-)	0.670
T2	5	29 (9.67–48.32)	
T3	40	21 (0.00–42.69)	
T4	13	10 (0.00–25.27)	
**Nodal stage**			
N0	32	29 (12.40–45.60)	0.505
N1	15	12 (3.16–20.84)	
N2	12	13 (0.00–31.67)	

Mismatch repair deficient patients (MMRD, *n* = 9) had a median OS of 12 months (95% CI: 6.15–17.84), while those with mismatch repair stable (MMRS, *n* = 50) had a median OS of 21 months (95% CI: 8.14–33.85). This difference was also not statistically significant (*p* = 0.692; Table 3, Figure 2A).

The median OS for patients with positive LC3β expression (*n* = 18) was 39 months (95% CI: 18.21– 59.79), compared to 19 months (95% CI: 10.24–27.76) for those with negative LC3β expression (*n* = 41). The difference in survival between these groups was not statistically significant (*p* = 0.335; Table 3, Figure 2B).

Likewise, the difference in survival based on tumor stage (*p* = 0.67) and nodal stage (*p* = 0.505) was not statistically significant (Table 3, Figures 2C, D).

Overall, MMR status, LC3β status, tumor stage and nodal stage did not show statistically significant differences in overall survival using the log-rank test.

## Discussion

Autophagy is an essential catabolic mechanism that serves important physiologic and survival functions in normal cells. In malignant cells however, autophagy has been associated with promotion of cell survival, metastasis, drug resistance and tumor recurrence all of which are associated with poor prognosis (6, 7). It is therefore a potential candidate as a target for cancer therapy as well as a prognostic marker. Its utility as a biomarker while not established has been explored in different tumors. Increased autophagy has been associated with worse prognosis in breast, esophageal, hepatocellular, and gastric carcinomas (3, 23–26). The aim of our study was to find out the expression in colorectal carcinoma of autophagy related protein LC3β using immunohistochemistry and establish the association with clinical-pathological characteristics and survival.

In our study, LC3β expression was described as positive or negative depending on the intensity and proportion of staining as previously described (21). The frequency of tumors which were positive was 36% (41/114) and was significantly more common in females. Other studies have shown different frequencies of LC3β expression in colorectal carcinoma. Wang et al., Shim et al., Choi et al., and Park et al. showed 86.5% (173/200), 46.5% (47/101), 74.7% (186/249), and 79% (119/151), respectively of LC3β expression in colorectal carcinomas (21, 27–29). This variation in frequency between the various studies may reflect the different scoring methods as there are currently no standardized scoring guidelines.

This study then aimed at establishing the association of LC3β expression with clinical pathological characteristics of colorectal carcinoma. There was no significant association between LC3β expression with age, tumor location, tumor pathological stage, tumor nodal stage, tumor grade, tumor budding, tumor infiltrating lymphocytes, and HER2 and mismatch repair protein status. Other studies have shown varying findings. Wang et al. (*n* = 200) and Park et al. (*n* = 151) found no association of LC3β expression with pathological tumor and nodal stage, age, gender and tumor grade (21, 28). In addition, Wang et al. showed no association with mismatch repair status. Shen et al. (*n* = 1,689) and Schimtz et al. (*n* = 128), however, found LC3β overexpression to be associated with high tumor grade (30, 31). The literature thus has contradictory findings on the frequency of LC3β expression and association with clinical pathological characteristics. Additional studies aimed at standardizing assessment and reporting are necessary for uniformity.

Our study then sought to establish the association of LC3β expression and survival. This was, however, hindered by lack of follow-up data in the records for most of the patients. Of the 114 subjects, only 59 patients had follow-up data updated in their records. This was thus a major limitation as far as meeting this objective was concerned. There was no significant difference in survival in relation to LC3β status. Schimtz et al. (*n* = 128) and Wang et al. (*n* = 200) found overexpression of LC3β to be associated with a worse overall survival while Choi et al. (*n* = 263) had contrary findings (21, 27, 31). Additional larger studies designed to investigate survival are necessary to determine the accurate position.

MMR status is an important biomarker as it has predictive and prognostic significance (32, 33). In our study, the frequency of MMR-deficient tumors was 14.9%. This is comparable to that reported in other studies. In a study carried out in the USA including 101,259 colorectal adenocarcinomas, the prevalence was 14.2% (34). A study conducted in South Africa showed found a frequency of 15% amongst Black patients and 12% amongst non-black patients (35).

MMR-deficient tumors were more common in males than women and were more common in patients < 60 years. This was, however, not statistically significant. Other studies have shown MMR-deficient tumor occur at a younger age (34). Most of the MMR-deficient tumors were in the right colon similar to what has been reported in other studies (36).

MMR deficient tumors were significantly associated with higher tumor- infiltrating lymphocytes (TILs). This finding is like what has been previously reported (36). While the association of MMR status and tumor and nodal pathological stage showed no significant difference, there were more patients with N0 disease in the MMR-deficient category. MMR-deficient tumors have been shown to be of a lower clinical stage at diagnosis and hence associated with a better prognosis (37).

HER2 overexpression was seen in 2.6% of the tumors. This is comparable to other studies which show low frequency of HER2 expression in colon cancer (38, 39). HER2 is a poor prognostic biomarker and predictive of response to specific HER2 antagonists (40).

In conclusion, our study found that LC3β is overexpressed in 36% of colorectal carcinomas and is more common in females. The LC3β status showed no significant association with age, gender, tumor location, tumor grade, tumor pathological stage, tumor nodal stage, tumor budding, tumor infiltrating lymphocytes, MMR status, and HER2 overexpression or patient survival. The frequency of mismatch repair deficiency and HER2 overexpression was 14.9% and 2.6%, respectively. To the best of our knowledge this is the only study on autophagy in colorectal carcinoma in Kenya. Additional larger studies designed to capture more accurate data on treatment and survival are indicated as autophagy is a promising prognostic and predictive biomarker.

## Data Availability

The raw data supporting the conclusions of this article will be made available by the authors, without undue reservation.
